# The clinical, endoscopic and histological spectrum of the solitary rectal ulcer syndrome: a single-center experience of 116 cases

**DOI:** 10.1186/1471-230X-12-72

**Published:** 2012-06-14

**Authors:** Shahab Abid, Ali Khawaja, Salima Ahmed Bhimani, Zubair Ahmad, Saeed Hamid, Wasim Jafri

**Affiliations:** 1 Section of Gastroenterology, Department of Medicine, Aga Khan University Hospital, Karachi, Pakistan; 2 Medical College, Aga Khan University Hospital, Karachi, Pakistan; 3 Department of Histopathology, Aga Khan University Hospital, Karachi, Pakistan

## Abstract

**Background:**

Solitary rectal ulcer syndrome (SRUS) is an uncommon although benign defecation disorder. The aim of this study was to evaluate the variable endoscopic manifestations of SRUS and its association with other diseases.

**Methods:**

All the patients diagnosed with SRUS histologically from January 1990 to February 2011 at The Aga Khan University, Karachi were included in the study. The medical records were reviewed retrospectively to evaluate the clinical spectrum of the patients along with the endoscopic and histological findings.

**Results:**

A total of 116 patients were evaluated. The mean age was 37.4 ± 16.6 (range: 13–80) years, 61 (53%) of the patients were male. Bleeding per rectum was present in 82%, abdominal pain in 49%, constipation in 23% and diarrhea in 22%. Endoscopically, solitary and multiple lesions were present in 79 (68%) and 33 (28%) patients respectively; ulcerative lesions in 90 (78%), polypoidal in 29 (25%), erythematous patches in 3 (2.5%) and petechial spots in one patient. Associated underlying conditions were hemorrhoids in 7 (6%), hyperplastic polyps in 4 (3.5%), adenomatous polyps in 2(2%), history of ulcerative colitis in 3 (2.5%) while adenocarcinoma of colon was observed in two patients. One patient had previous surgery for colonic carcinoma.

**Conclusion:**

SRUS may manifest on endoscopy as multiple ulcers, polypoidal growth and erythematous patches and has shown to share clinicopathological features with rectal prolapse, proctitis cystica profunda (PCP) and inflammatory cloacogenic polyp; therefore collectively grouped as mucosal prolapse syndrome. This may be associated with underlying conditions such as polyps, ulcerative colitis, hemorrhoids and malignancy. High index of suspicion is required to diagnose potentially serious disease by repeated endoscopies with biopsies to look for potentially serious underlying conditions associated with SRUS.

## Background

Solitary rectal ulcer syndrome (SRUS) is an uncommon benign disorder of defecation, the mechanism of which is poorly understood. Most accepted etiopathogenetic mechanism of SRUS is chronic mucosal and hypoperfusion induced ischemic injury to the rectal mucosa. This is associated with paradoxical contraction of the pelvic floor leading to mucosal prolapse and pressure necrosis of rectal mucosa [1,2]. Other hypothesis suggests that external anal sphincter produces abnormal pressure gradients in the opposite direction which results in abnormal defecation leading to SRUS [2].

Due to wide range of clinical symptomatology and endoscopic findings, SRUS may often simulate other disorders such as inflammatory bowel disease (IBD) and neoplasms [3-5]. SRUS commonly manifests as bleeding or mucoid discharge per rectum associated with abdominal pain, straining, rectal prolapse and sensation of incomplete evacuation [2,6,7]. Endoscopic findings insinuate that the term ‘SRUS’ is a misnomer since neither are the lesions always solitary nor are they always ulcerative. Furthermore, they can affect regions other than the rectum [6-8]. Histological analysis is considered to be the cornerstone for diagnosing SRUS with ‘fibromuscular obliteration’ being the characteristic finding [9]. SRUS is known for its chronic course and a definitive guideline for management remains disputed.

SRUS forms a component of the spectrum of benign defecation disorders comprising rectal prolapse, proctitis cystica profunda (PCP) and inflammatory cloacogenic polyp; the four entities sharing the clinical and pathological features. The characteristic histological finding of fibromuscular obliteration of SRUS along with other histological findings including glandular crypt abnormalities, thickened and splayed muscularis mucosa, surface ulceration, hyperplastic and serrated mucosa, mucous cell proliferation and dilatation of glands have been found to be overlapping in these four entities [10-14]. Such findings are more commonly seen in patients with features of overt or occult mucosal prolapse leading to polypoidal mucosa. Therefore, it has been proposed that the four entities should be grouped under the term of “mucosal prolapse syndrome” [10-13].

The data on clinical and endoscopic spectrum of SRUS is scarce in this region of the world. Therefore, we carried out this study to deduce the clinical and endoscopic spectrum in patients with SRUS in our setting. We are presenting the clinical and endoscopic spectrum of SRUS patients who presented to our hospital in the last twenty-one years.

## Methods

The medical record numbers of patients diagnosed with SRUS from 1990 to 2011 were retrieved from the electronic database of The Aga Khan University, Karachi, Pakistan and reviewed retrospectively. We analyzed only those patients whose complete records (clinical notes, endoscopic findings and histology) were available.

Diagnosis of SRUS was based on characteristic endoscopic and histological findings. Lesions on endoscopic findings were divided on the basis of numbers as solitary or multiple and on the basis of appearance as ulcerative, polypoidal/nodular or erythematous mucosa only. The histological criteria included splaying of smooth muscle cells and fibrosis of the lamina propria leading to fibromuscular obliteration, surface ulceration, crypts and mucosal gland distortion and hyperplasia which may lead to polypoidal appearance. Acute inflammation referred to the presence of neutrophils in the lamina propria and glands while chronic inflammation was characterized by lymphoplasmacytic infiltrate in the lamina propria. Hemoglobin level less than 11 g/dl was defined as anemia.

The recorded data was compiled on the SPSS version 17.0 and frequency analysis was performed. Continuous variables are presented as mean ± standard deviation and categorical variables are presented as number of patients and percentages in parenthesis.

Ethical approval was taken by the ethics review committee of the Aga Khan University Hospital prior to commencement of the study.

## Results

A total of 116 cases were evaluated for clinical, endoscopic and histological findings that had the complete information available in their medical records. Mean age of the group was 37 years (range 13–80 years); males were 61 (53%).

The most common symptom was bleeding per rectum affecting 95 (82%) patients followed by abdominal pain (49%), constipation (23%), diarrhea (22%) and mucus per rectum (17%). Manual digital evacuation was reported in 8 (7%) patients.

**Figure 1:**
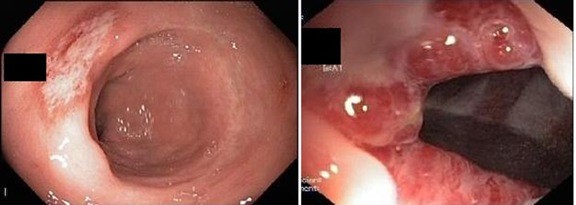
**(Top) A superficial solitary ulcer in the rectum.** This 36 year old lady presented with bleeding per rectum (BPR), abdominal pain, diarrhea and tenesmus. (Bottom) A column of congested internal hemorrhoids in the same patient. The symptoms improved with psyllium husk and banding of hemorrhoids.

**Figure 2:**
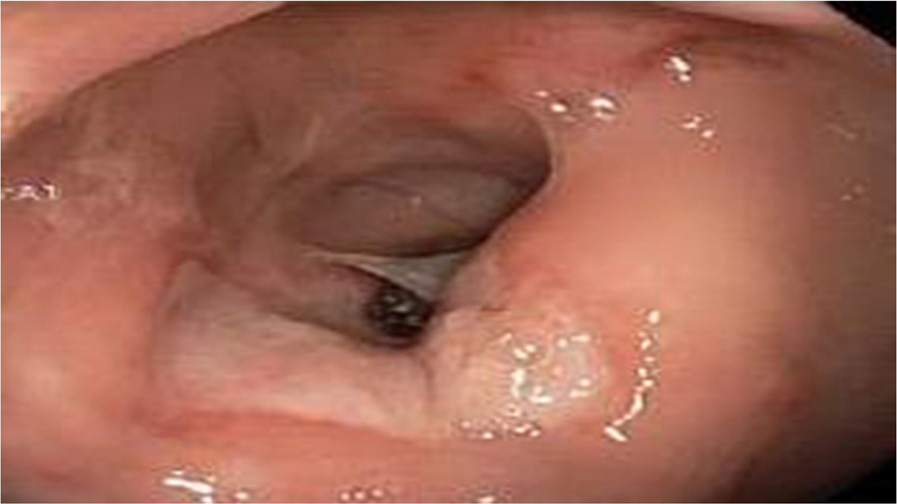
**Solitary rectal ulcer syndrome as multiple ulcerative lesions.** This 55 year old gentleman reported manual digital evacuation of feces.

**Figure 3:**
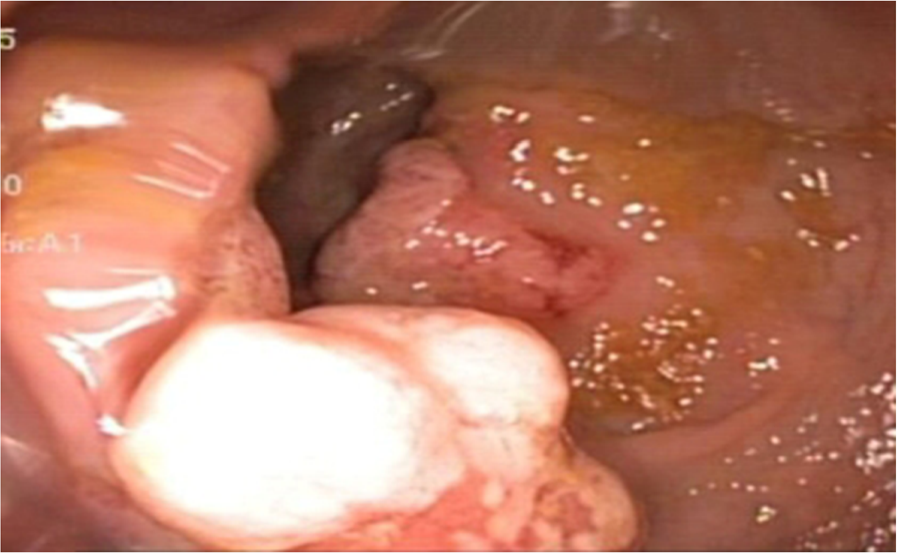
**Solitary rectal ulcer syndrome as a large polypoidal growth in a young boy.** He presented with rectal bleeding. Large circumferential growth was seen on follow-up endoscopy. Anorectal excision was performed for cure of this patient.

**Figure 4:**
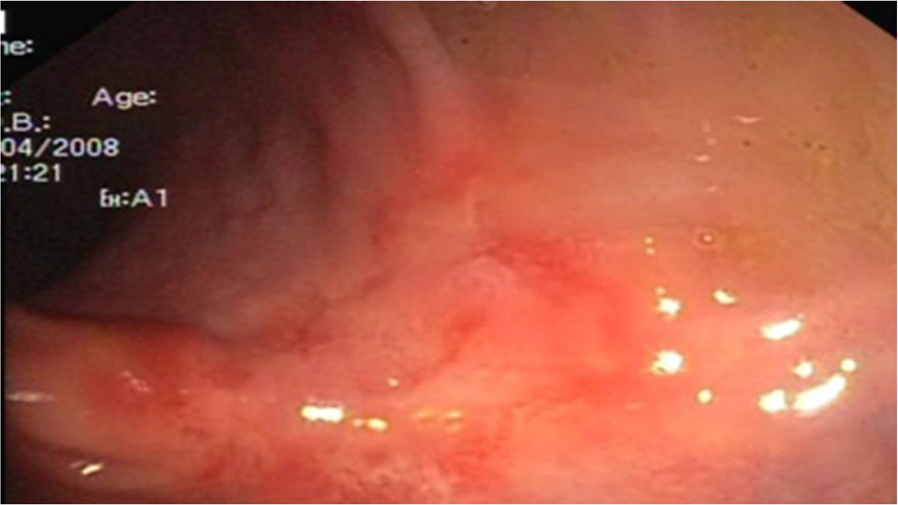
Solitary rectal ulcer syndrome as erythematous rectal mucosa.

**Figure 5:**
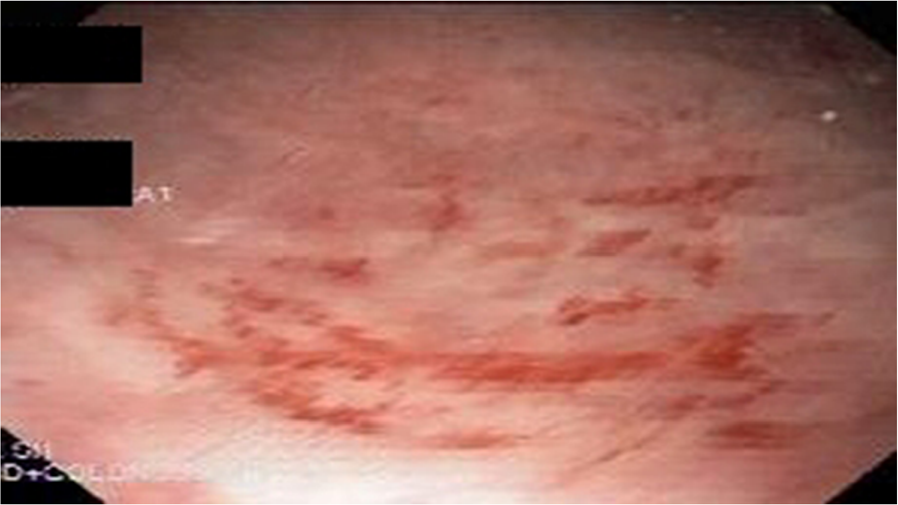
**Solitary rectal ulcer syndrome as multiple telengectatic spots in a 21 year old woman.** She presented with rectal bleeding and anemia. She was started on bulk laxatives and mesalazine enema to which she did not respond, argon plasma coagulation was performed. Follow up endoscopy showed a healed ulcer and congested internal hemorrhoids. Band ligation was performed which resulted in resolution of symptoms.

Endoscopic findings revealed solitary (Figure 1) and multiple lesions (Figure 2) in 79 (68%) and 33 (28%) patients, respectively. On the basis of appearance, 90 (78%) of the lesions were ulcerative (Figure 1) while 29 (25%) were polypoidal/nodular (Figure 3). Three (3%) patients had erythematous mucosa only (Figure 4) while one patient had telengectatic spots (Figure 5).

The clinical presentation and endoscopic findings are summarized in Tables 1 and 2 respectively.

**Table 1:** Symptomatology of patients with Solitary rectal ulcer syndrome (n = 116)

**Presenting symptoms**	**Number of patients (%)**
**Bleeding per rectum**	95 (82)
**Mucus per rectum**	20 (17)
**Abdominal pain**	57 (49)
**Straining at stool**	36 (31)
**Rectal Prolapse**	13 (11)
**Constipation**	27 (23)
**Diarrhea**	26 (22)
**Tenesmus**	07 (06)
**Altered bowel habits**	12 (10)
**Sensation of incomplete evacuation**	05 (04)
**Anemia**	26 (22)
**Perianal pain**	16 (14)
**Weight loss**	18 (16)
**Digital evacuation**	08 (07)
**Asymptomatic (incidental finding)**	02 (02)
**History of Ulcerative colitis**	03 (2.5)
**History of Adenocarcinoma**	02 (02)
**History of surgery for Adenocarcinoma**	01 (01)

**Table 2:** Endoscopic Findings of patients with Solitary Rectal Ulcer Syndrome (n = 116)

**Endoscopic findings**	**Number of patients (%)**
**Solitary lesion**	79 (68)
**Multiple lesions**	33 (28)
**Ulcerative**	90 (78)
**Polypoidal/nodular**	29 (25)
**Erythema only**	03 (03)
**Telengectatic spots**	01 (01)
**Hemorrhoids**	07 (06)
**Hyperplastic polyps**	04 (3.5)
**Adenomatous polyps**	02 (02)

**Figure 6:**
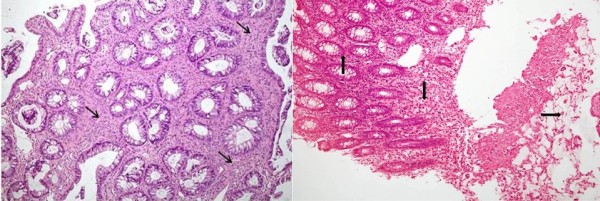
**An ulcerated, friable mass was found on endoscopy in the upper rectum along with a polyp in a 21 year old gentleman.** Polypectomy was performed. The biopsy of the mass and polyp showed SRUS and infiltrating colorectal adenocarcinoma, respectively. A repeat biopsy from the mass after one week revealed an infiltrating colorectal adenocarcinoma with signet cells instead of SRUS. He had uneventful recovery from high anterior resection and chemotherapy. (Top) Photomicrograph of first biopsy showing rectal glands separated by splaying smooth muscle fibers and fibrosis (arrows). H&E x 20. (Bottom) Photomicrograph of the second biopsy showing normal large bowel mucosa on the left (↑), mucinous areas on the right (→) and signet areas in the center (↓). H&E x 10.

Associated underlying conditions were hemorrhoids in 7 (6%) (Figure 1), hyperplastic polyps in 4 (3.5%), adenomatous polyps in 2 (2%) and adenocarcinoma of colon in two patients (Figure 6) while 3 patients had history of ulcerative colitis. One patient had previous surgery for colonic carcinoma.

Histological examination showed fibromuscular obliteration in all, surface ulceration in 68 (59%) while crypts distortion was reported in 20 (17%) patients. Chronic inflammatory infiltrates were seen in 38 (33%) patients (Table 3).

**Table 3:** Histological findings of Solitary rectal ulcer syndrome (n = 116)

**Histological findings**	**Number of patients (%)**
**Fibromuscular obliteration**	116 (100)
**Surface Ulceration**	68 (59)
**Crypts distortion**	20 (17)
**Mucosal glands distortion**	27 (23)
**Crypts hyperplasia**	12 (10)
**Inflammation:**	
1. Acute	05 (04)
2. Chronic	38 (33)
3. Both	20 (17)
4. None	53 (46)

## Discussion

SRUS is a chronic disorder which can present with diverse endoscopic findings. Since diagnosis on the basis of clinical symptoms alone is difficult, it is imperative for the clinicians to keep this entity in their differentials on endoscopic examination to reach the correct conclusion. Incidences of under and misdiagnosed cases have been reported in literature [3,15-17]. Much of the lapse in diagnosis is ascribed to the lack of familiarity of clinicians with endoscopic revelations and the actual condition associated with SRUS. A typical solitary rectal ulcer is a shallow based ulcerating lesion encircled by hyperemic mucosa [8,18]. This study to the best of our knowledge is the largest series of patients with SRUS. It met with intriguing diversity in the appearance of these lesions from being plain ulcerative to polypoidal and from presenting as an erythematous mucosa to multiple ulcerative lesions. Other interesting findings included multiple telengectatic bleeding spots (Figure 5) and a large lesion lying in close proximity of a cancerous rectal polyp (Figure 6).

The findings in the present series correspond with the literature in terming SRUS as a misnomer. The polypoid or nodular variant of SRUS has a higher tendency to be misdiagnosed and confused with other presentations for instance those of inflammatory polyp, hyper plastic polyp or rectal carcinoma [3,19]. Hence, variability with which SRUS presents on endoscopy is more profound than is generally comprehended.

Histopathological analysis forms the cornerstone of diagnosing SRUS and is a requisite to rule out any other underlying disease. In contrast to the inconsistency and discrepancy on clinical and endoscopic findings, histological characteristics associated with SRUS are well documented. Key histological features encompass fibromuscular obliteration of the lamina propria with splaying of muscularis mucosae upward between the crypts, thickened mucosa and glandular distortion [9,20]. These features are also seen to be overlapping in other benign defecation disorders including rectal prolapse, PCP and inflammatory cloacogenic polyp [10-13]. Our study revealed fibromuscular obliteration in all patients, with 59% additionally having surface ulceration. Other findings such as mucosal glands and crypts distortion were less documented. In contrast, a study documented all 13 patients having crypts distortion and surface serration [2]. Moreover, no inflammation was seen in majority of the lesions in our series and when present, inflammatory infiltrates were usually found to be mild in character. Nonspecific histological findings may be apparent on SRUS lesions which include hyperplastic and distorted crypts together with epithelial atypia and high degree of inflammation [18]. These findings along with similar symptomatology and variable endoscopic findings can make it challenging at times to differentiate SRUS from IBD.

Three patients had a history of ulcerative colitis in the current series. Development of SRUS in patients with past history of ulcerative colitis can lead to confusion whether the patient’s symptoms are due to an exacerbation of the primary disease or because of SRUS. In another study, seven patients of SRUS were at first misdiagnosed for IBD [3] while cases of SRUS in patients with history of ulcerative colitis have also been reported [21,22]. However, fibromuscular obliteration and excess mucosal collagen helps in differentiating SRUS from IBD on morphological analysis [23]. ‘Diamond shaped crypts’ have also been seen to be an important finding in diagnosing SRUS. A study comprising a cohort of 32 patients with SRUS revealed diamond shaped crypts in all the patients as compared to only one case of IBD while another study reported over half the patients having this feature on histological analysis [3,24].

Studies emphasize that histopathology of SRUS may be associated with a deeper concealed malignancy [25,26]. In one of the study it was documented that malignant tumors might present with histological findings suggesting SRUS initially and later develop characteristics of malignancy which suggests SRUS has the potential to progress to malignancy [25]. Another study reemphasizes this aspect by demonstrating loss of hMLH1 gene expression in several cases of SRUS indicating the possibility of neoplastic progression [27]. A case of well differentiated infiltrating adenocarcinoma in the focus of SRUS has also been reported and the authors speculated that there is a chance of adenocarcinoma originating from SRUS mucosa [26]. These observations from the available literature support findings in one of the case (Figure 6) in our series. However, there could be a possibility of missing the neoplastic lesion initially in our case when the biopsy was taken the first time but it may reflect the simultaneous existence of SRUS with adenocarcinoma so it is emphasized to have a high index of suspicion and repeated examinations with multiple biopsies to be taken. Similarly, two other patients in present series demonstrated coexistence of SRUS with adenomatous polyps. However, it should also be emphasized that the neoplastic lesions of adenoma and adenocarcinoma have been reported as associations only with SRUS and that no causal relationship has been established yet from the data in the present literature.

Inconsistency in morphologic appearances of associated lesions increases the likelihood of delayed or erroneous diagnosis of SRUS [3,4]. Our series depicted that rectal bleeding and abdominal pain were the most common complains. Large number of patients also complained of constipation and/or diarrhea while mucus per rectum and perianal pain were encountered less frequently. This corresponds to other studies published with similar complaints [2,7,20]. BPR occurs most likely due to ulcerations or direct trauma to the mucosa. Manual digital evacuation is the other important factor causing direct injury to the rectal mucosa and possibly the cause of bleeding in SRUS [28]. In our study, only eight patients were documented to perform rectal digitations. This low number of patients with history of digital evacuation of feces may be due to the retrospective nature of the data (poor documentation) or hesitation on the part of the patients in revealing it to the physician or unwillingness to have them documented in records. Another important point is the time span between onset of symptoms and establishment of a correct diagnosis in patients with SRUS which range from three months to 30 years [3]. The time that elapses during this period might have important clinical consequences like weight loss and anemia as demonstrated in the present series that 22% of patients presented with anemia and 16% had weight loss at the time of diagnosis.

## Conclusion

Present study reveals that the rectal bleeding, abdominal pain and constipation were the most common symptoms encountered in patients with SRUS. Ulcerative lesions remain the most common observation on endoscopy but lesions other than ulcerative including polypoidal/nodular and erythematous mucosa were also present. The clinical and pathological features of SRUS are found to be shared by other bengin defecation disorders which reflect diverse manifestations collectively grouped as mucosal prolapse syndrome. SRUS may also be found coexisting with polyps, ulcerative colitis, hemorrhoids and malignancy.

A high index of suspicion is therefore required to diagnose potentially serious disease by repeated examinations and biopsies for histopathology.

## Competing interests

The authors declare no conflict of interest and approve the final version of the manuscript.

## Authors’ contributions

SA contributed in terms of original idea, study design, protocol writing, supervising the project and critically editing the manuscript. AK and SAB contributed in protocol writing, data collection, data entry, data analysis and manuscript writing. ZA reviewed the histopathology slides and final manuscript. SH and WJ contributed their patients and in manuscript writing. All authors have read and approve the final version of the manuscript.

## Pre-publication history

The pre-publication history for this paper can be accessed here:

http://www.biomedcentral.com/1471-230X/12/72/prepub
